# Recovery and Youth: An Integrative Review

**DOI:** 10.35946/arcr.v40.3.06

**Published:** 2020-12-17

**Authors:** Andrew J. Finch, Jordan Jurinsky, Billie May Anderson

**Affiliations:** Department of Human and Organizational Development, Vanderbilt University, Nashville, Tennessee

**Keywords:** recovery, substance-related disorders, alcohol-related disorders, adolescence, continuum of care, alcohol, youth, recovery capital

## Abstract

Although rates of alcohol and other substance use disorders in adolescents have been estimated for decades, little is known about the prevalence, pathways, and predictors of remission and long-term recovery among adolescents. This article provides an integrative review of the literature on youth recovery. A final selection of 39 relevant articles was grouped into five sections: treatment outcomes, special emphasis populations, recovery-oriented systems of care, families, and non–abstinence-based approaches. The review recommends more adolescent research in three basic areas: more research about medication-assisted treatment and recovery as well as harm reduction approaches for adolescents; expansion of research on recovery practices for youth who do not receive treatment due to personal choice or societal disparities; and more life course research, which may begin with adolescent participants and extend across the life span. Additionally, the authors suggest the recovery capital model for adolescents and the neuroscience of addiction may provide additional precision and direction for the study of youth recovery.

## INTRODUCTION

Recovery from alcohol use disorder (AUD) or other substance use disorder (SUD) is an evolving concept. This article reviews youth recovery, as little is known about the prevalence, pathways, and predictors of remission and long-term recovery among adolescents and how these may contrast with recovery in emerging and older adults. Although much of the literature on alcohol or other substance use in youth has focused on prevention, adolescents can and do develop AUD or other SUD. Data reported by the annual National Survey on Drug Use and Health showed nearly 1 million youths (ages 12 to 17) needed treatment for AUD or SUD in 2018, although only 83,000 of them received services in a treatment center.^1^

Historian William White has suggested that the recovery movement began in the late 1990s with an extraordinary cultural and political mobilization supported by the Recovery Community Services Program of the Substance Abuse and Mental Health Services Administration’s (SAMHSA) Center for Substance Abuse Treatment.^2^ White identified the 2001 Recovery Summit in St. Paul, Minnesota, which launched Faces and Voices of Recovery, as a milestone in the recovery advocacy movement. The recovery movement impacted research literature as well. Kaskutas, Witbrodt, and Grella conducted a Google Scholar search dating to 1959 and found a significant increase from 2001 to 2012 in the number of articles about alcohol or other substance use with “recovery” in the title.^3^ The American Psychiatric Association then released the fifth edition of its *Diagnostic and Statistical Manual of Mental Disorders* (DSM-5) in 2013, which revised the diagnostics for SUD, creating a range of symptoms from mild to moderate to severe. This revision helped shift the perception of SUD as existing along a continuum of severity rather than as a distinct positive or negative diagnosis, which was intended to impact how practitioners treated SUD and how researchers studied it.

In the midst of the burgeoning recovery advocacy movement, SAMHSA’s Center for Substance Abuse Treatment convened the first National Summit on Addiction Recovery in 2005 to create one of the first definitions of recovery: “Recovery from alcohol and drug problems is a process of change through which an individual achieves abstinence and improved health, wellness, and quality of life.”^4^ In 2011, SAMHSA broadened this definition even more by removing the mention of abstinence as a criterion for recovery: “a process of self-directed change through which individuals improve their health and wellness, live self-directed lives, and strive to reach their full potential.” These were only two of nearly a dozen definitions to come. According to Ashford and colleagues, at least 10 relevant definitions of recovery emerged between 2005 and 2017, from which their own Recovery Science Research Collaborative (RSRC) highlighted three as the “leading definitions of recovery”: SAMHSA in 2011, the American Society of Addiction Medicine in 2013, and the Betty Ford Institute Consensus Panel in 2007.^5^^(p180)^ Guided by these statements, the RSRC crafted its own definition: “Recovery is an intentional, dynamic, and relational process that involves sustained efforts to improve multiple aspects of wellness, and which may vary by individual, social, and experiential contexts.”^5^^(p183)^ In an effort to be more holistic and inclusive, similar to SAMHSA’s 2011 description, the RSRC made no mention of reducing or abstaining from alcohol or substance use.

Along the same lines, none of the major efforts to conceptualize recovery have specified age or developmental concerns, which creates the impression that either the definitions were intended for adults, or the drafters considered adolescent recovery to be indistinguishable from adult recovery. In most instances, youth recovery simply has not been addressed in the definitional literature. Over the last decade, however, addiction neuroscience has shown that alcohol or other substance use during adolescence has a substantial impact on brain development. According to the National Institute on Drug Abuse: “The fact that this critical part of a teen’s brain [the prefrontal cortex] is still a work in progress puts them at increased risk for trying drugs or continuing to take them. Introducing drugs during this period of development may cause brain changes that have profound and long-lasting consequences.”^6^^(p10)^ In addition, youths under age 18 cannot legally drink alcohol without parental supervision or use cannabis in states where recreational use is allowed, must be enrolled in school, and are considered minors and thus legally dependent on parents or guardians. For youths in recovery, therefore, the developmental, legal, and familial context fundamentally differs in ways that render adult-based conceptualizations of recovery insufficient.

Adolescent treatment and recovery support programs expanded at the same time as definitions of recovery were being adopted, and the youth data from both the annual National Survey on Drug Use and Health and the Monitoring the Future studies have shown precipitous drops in virtually every indicator of alcohol or other substance-related disorder—including youth meeting the criteria for SUD, youth needing treatment, and youth receiving treatment. The number of youths ages 12 to 17 who needed treatment—a key indicator of potential referrals—was nearly 2.3 million in 2002, but by 2018, the number had fallen to 946,000.^1^

The reason for the decline in adolescents with SUD is uncertain, but the recovery movement no doubt played a role by spurring programs that reduced recidivism and provided tertiary prevention. AUD and SUD, though, have persisted, as have the treatment and recovery support gaps. Despite the efforts to define and potentially quantify the recovery process, the specific phenomenology of youth recovery has remained diffuse. Although substantial literature on adolescent AUD and SUD and treatment outcomes has arisen over the last 20 years, this review of the youth recovery literature has been complicated by inconsistent conceptions of the ages bounding “youth”; the definition, genesis, and life course of adolescent recovery; and the outcomes that are deemed successful. There exists a tapestry from which to divine an understanding of adolescent recovery, but a coherent typology has been elusive. This article thus uses other topics in the Recovery From AUD featured topic series as an organizational guide. As most of the issue’s subtopics are not exclusively youth-focused, this article brings adolescence to the forefront, discussing (1) treatment outcomes, (2) special emphasis populations, (3) recovery-oriented systems of care, (4) families, and (5) non–abstinence-based approaches. This article concludes with a call for a clearer and more focused definition of recovery from AUD and SUD for adolescents, as well as more prospective and longitudinal research on sustained recovery and its impact on individual young people and society.

## METHODS

This article provides a thorough and current review of the literature supported by representative references, utilizing an integrative review approach.^7^ The methodology reflects the topic series’ guidelines to review AUD among youth with a focus on recovery and within a limit of 50 references. Having three authors minimized potential bias, and each person conducted an independent review of articles. Multiple meetings were held discussing search criteria, findings, and selection. The process was emergent, iterative, and reflexive, and it considered prior reviews looking at similar issues. The authors ultimately decided the best organizing frame was from the topic series itself. Other frames emerged and were considered, but the topics from the journal itself ultimately worked best for consistency and clarity.

### Problem Identification

This review was initially conceived as an exploration of the prevalence, pathways, and predictors of remission and long-term recovery among not only adolescents, but also emerging adults, commonly understood as the population ages 18 to 25. It also was intended to address not only recovery support services but also early interventions. After multiple conceptual discussions and after receiving consent from the editors, the authors agreed to focus on youths ages 12 to 18, the life phase usually referred to as adolescence. The literature and prevalence data on emerging adults (ages 18 to 25) are robust and worthy of their own review, but including that age group in this review could have drowned out the focus on adolescents. Although the life phase of transitional-age youth (ages 16 to 24) includes minors and youths transitioning from state custody and foster care, including that entire group also necessarily adds emerging adults, thus creating similar issues. As the adolescent age group is fundamentally different from emerging adults in a number of ways, including legal status, brain development, recovery capital, and family involvement, the authors felt a study of the trends and gaps in the literature on adolescents was needed. The scope also was narrowed to focus on the recovery process rather than the early intervention and treatment outcome literature highlighting specific treatments (such as multidimensional family therapy or motivational interviewing). This allowed the review to approach recovery as part of the treatment process as well as distinct from it. As treatment was not the focus of this review, the only treatment articles considered for this review incorporate investigations into specific factors that influence the recovery process. Treatment studies exploring treatment outcomes and/or effectiveness per se were considered beyond the scope of this review.

### Literature Search

Articles were included if they explored problematic alcohol and drug use or AUD and recovery among adolescents. As the adolescent life phase is understood differently in the literature— sometimes containing 18-year-olds and sometimes not—this review included articles focused on people age 18 and younger. Articles were included if they explicitly mentioned recovery or expanded on facets of the recovery process, such as personal or environmental characteristics that support recovery, broadly defined. Such topics included abstinence, sobriety, mutual aid, relapse, and alternative peer groups. Studies were excluded that focused solely on treatment outcomes, screening, or prevention. The year of publication was not considered when determining eligibility.

**Figure 1:**
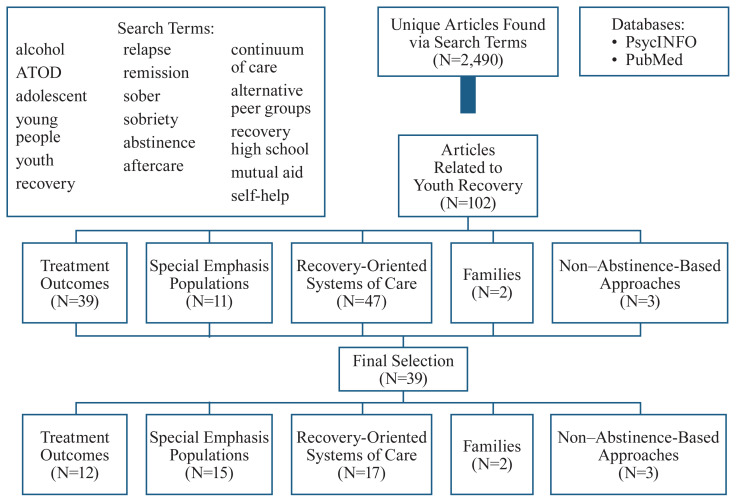
Literature search tree for an integrative review of recovery and young people. *Note:* ATOD, alcohol, tobacco, and other drugs.

A systematic search was conducted in November 2019 of published studies in PsycINFO and PubMed (see Figure 1). These databases represent curated repositories of health, social science, and medical/clinical literature. Databases were searched for major themes of alcohol and recovery among adolescents. Based upon journal guidelines, articles must have explicitly included alcohol use in order to be considered for the study. Due to the conceptual ambiguity of recovery, additional terms commonly used in the field over the past few decades were included: relapse, remission, self-help, sobriety, and abstinence. Targeted searches also incorporated the key words “alternative peer group” and “recovery high school.” After the removal of duplicates, the search resulted in 2,490 unique articles (specific search strings available upon request).

### Data Evaluation

Two authors independently reviewed half of the articles’ titles and abstracts, and the lead author randomly reviewed articles for fidelity. In the initial screening, the full text of any ambiguous article was reviewed by multiple authors until a consensus was reached. After screening, 102 articles were identified as relating to youth recovery.

### Data Analysis

Authors independently reviewed the 102 articles identified to create broad categories based on the variables and/or context studied (e.g., mutual aid, adolescent peer group, relapse). Due to the complexity and breadth of the literature, authors independently reviewed and coded articles for key themes and identified one to two main foci. The authors then met and refined the list of key themes. With a unified list of foci, authors again reviewed and coded articles. More than 20 major topics and 53 subtopics were identified. Because of the limited space and the range of topics, authors chose to organize the major topics to mirror those covered in the topic series. The 23 primary categories were thus grouped into five sections for review: treatment outcomes, special emphasis populations, recovery-oriented systems of care, families, and non–abstinence-based approaches. The description and rationale for each of those sections is discussed earlier.

After reaching an agreement on the conceptual framework, two authors independently identified which of the 102 articles to include in the literature review. This process included assessing articles on individual characteristics as well as considering the breadth of articles reviewed. Individual study characteristics included sample size for quantitative studies, credibility enhancements such as triangulation in qualitative work, publication year, recovery focus, and implications of findings. Macro-level considerations included representing a range of authors, study designs, distribution across topic areas, and conceptual frames. Upon completion, those two authors met to reach a consensus, and the lead author then independently assayed the articles to approve of the final selection of 39 for inclusion, a number within the journal’s preferred limit of 50 total citations (Table 1).

## RECOVERY AS A TREATMENT OUTCOME

Until relatively recently, adolescent recovery from AUD or other SUD has been researched mostly as part of a linear model of addiction treatment. Recovery was understood to be abstinence-based, and adolescent recovery usually was assumed to include some form of treatment. Indeed, most researchers have viewed adolescent recovery as the result of successful treatment rather than a distinct phenomenon. If recovery programs were studied at all, they were seen as aftercare, or continuing care, to sustain the gains of treatment. Articles examining treatment outcomes and relapse thus account for the majority of the articles about recovery and youth. Treatment outcomes (e.g., abstinence, symptom reduction) were identified traditionally as the dependent variable, as opposed to the growing body of research studying recovery itself as the dependent variable. Instead of viewing recovery as its own construct, the following articles represent those studies that evaluated treatment outcomes as a proxy for recovery.

Treatment outcome articles cover myriad modalities, including both specialty (i.e., treatment centers, hospitals) and non–specialty treatment (i.e., doctor’s offices, emergency rooms, support groups). Within the context of recovery from a treatment lens, longitudinal treatment outcome studies provide insight into adolescents’ behavior post-treatment and the variables that impact abstinence or relapse. For the purpose of this review, articles researching treatment modalities were included if they focused on treatment in a recovery context. This means the study emphasized how the recovery process supported treatment instead of whether a singular treatment modality was effective, with the locus being the aspects of recovery rather than the components of treatment.

There is much research evaluating potential mediators and moderators of treatment outcomes, such as social skills and cognitive abilities. Brown and colleagues, for example, studied adolescents’ behavior for 4 years post-treatment, and their findings elucidate variables impacted by the developmental transition from adolescence into young adulthood, which may uniquely impact treatment outcomes.^8^ Other literature explored internal factors, such as coping skills, developmental and neurocognitive considerations, and psychosocial factors.^9^–^11^

Due to the social and environmental pressures faced by adolescents, the development of positive psychosocial skills can be an essential element in treatment, as such skills have been associated with avoiding relapse.^9^,^11^ From a developmental perspective, coping skills and neurocognitive abilities were found to distinctively impact adolescents’ relapse.^12^ These factors were more salient for adolescents with lower intellectual abilities, whereas other factors may be more salient for those with average or above-average intellectual abilities.^12^ According to Latimer and colleagues, an adolescent with at least one protective factor (e.g., social connectedness, goal directedness, peer abstinence), who completed long-term treatment followed by continuing care, was more likely to achieve successful outcomes compared to those with fewer protective factors.^11^

External factors, such as one’s environment or social influences, can also impact treatment outcomes. Peer affiliation and influence have been shown to play critical developmental roles in adolescents’ post-treatment behaviors. When adolescents return to their previously held social groups and support systems following treatment, they can be faced with contradicting desires to abstain from alcohol and other substance use while simultaneously maintaining their relationships with substance-using peers.^13^ Among adolescents who relapsed post-treatment, Cornelius and colleagues found social pressure, withdrawal, and negative affect to be the most common factors.^14^

Continuing care has been highlighted in the literature as supporting treatment gains and preventing relapse. Kaminer and Godley suggested that, because adolescents were less likely than adults to remain abstinent after one treatment episode, evaluating continuing care was essential.^15^ Cavaiola and colleagues highlighted the importance of continuing care as part of the recovery process in an early article published 30 years ago.^16^ While still emphasizing abstinence and relapse prevention, Cavaiola et al. evaluated an array of factors impacting post-treatment continuing care among adolescents to provide a more holistic view of recovery, including integration into mutual aid, relapse prevention and relapse management, relationships, resistance and denial, grief and loss issues, self-esteem issues, family treatment issues, and dual diagnosis.^16^

The complex nature of recovery has led to divergence in how researchers have approached relapse and abstinence for youth. It is critical to note the discrepancies in definitions of “relapse” and the subsequent impact on the evaluation of treatment outcomes and recovery for young people.^17^ Relapse and relapse prevention are multifaceted phenomena closely associated with treatment outcomes; yet, the field has been moving away from seeing recovery as requiring abstinence. Chung and associates, for example, implemented a trajectory analysis to demonstrate how a return to use does not necessarily indicate an adolescent is not in recovery or reducing their problematic behavior.^18^ As of late, the nascent body of literature dedicated to harm reduction has highlighted the differences between abstinence, reducing use, and using less harmful substances as the dependent variables in research studies. Although there have been few studies of harm reduction for youths, Kaminer and colleagues found that the relationship between abstinence as a post-treatment goal and long-term success is stronger than if the goal is harm reduction.^19^ A substantial number of studies have been designed through a treatment outcome lens, which defaults to “recovery” if an adolescent is abstinent. In essence, for youth, recovery has been studied more as an emergent latent variable than as its own designated entity.

## SPECIAL EMPHASIS POPULATIONS

Differences in relapse and relapse prevention among subpopulations of adolescents form a subset of the literature viewing adolescent recovery through a treatment outcome lens. The recovery process post-treatment had a different trajectory based upon various factors, such as the intersectionality of an adolescent’s recovery and cultural identity, including gender, race, and/or ethnicity. Populations highlighted here include students and adolescents with co-occurring disorders or traumatic experiences.

Although evaluating co-occurring disorders in adolescence can be problematic due to diagnostic criteria that often exclude people under age 18, there is a small body of literature that studies the impact of psychiatric comorbidity on relapse and treatment outcomes. Psychiatric symptoms have been found to influence post-treatment relapse among adolescents with AUD or other SUD and a co-occurring Axis I diagnosis.^20^ Sterling and colleagues found engagement during treatment to be essential for adolescents with co-occurring disorders, because abstinence during the first year was associated with reduced substance use and symptoms of mental health disorders after 3 years.^21^ The authors suggested mental health symptomology should not be excluded when evaluating the treatment outcomes and recovery process of adolescents with co-occurring disorders, especially given that positive mental health outcomes during treatment were associated with long-term recovery benefits.^21^

Research evaluating the relationship between a high incidence of alcohol and other substance use for adolescents with trauma histories is growing, but the literature is still limited. The contribution or impact of lifetime trauma on an adolescent’s substance use or on the treatment process has been studied, but how trauma relates to an adolescent’s recovery has not been examined. For example, the relationship between social anxiety disorder and lifetime trauma, as studied by Pagano and colleagues highlighted the indirect influence of trauma on peer support systems and boundary setting in the treatment process.^22^

Similar to other subpopulations, the prevalence of alcohol or other substance-related disorders for adolescents based on gender, race, and/or ethnic identity has been studied at length. Limited literature, however, is available to explain the impact of these identities on recovery. Research has evaluated post-treatment behaviors that have been impacted by an adolescent’s culture. For example, although there are differences in spirituality and religiosity levels between Black and White adolescents receiving treatment for AUD or other SUD, the findings suggested that religiosity was a predictor of 12-step-related behaviors but not of treatment outcomes.^23^ In the same study, a significant gender disparity was found in that women were more likely to take the actions outlined in the 12 steps.^23^

Another unique consideration for this age group is the status of student. As most states require people under age 18 to be enrolled in school, studies have not compared recovery processes for student versus nonstudent adolescent samples. There is little research, though, studying the impact of recovery on young people’s academic outcomes. In one such study, a neuropsychological test battery evaluating five key domains was used as a proxy for academic outcomes by evaluating cognitive functioning.^24^ During early abstinence from heavy episodic drinking, adolescents’ prospective memory, cognitive switching, inhibition task accuracy, and visuospatial abilities developed significantly.^24^

It can be surmised that due to the relatively small number of adolescents in recovery, it could be prohibitively challenging to study sample sizes that result in statistically significant findings. Although prevalence of alcohol and other substance use among specific adolescent subpopulations, such as LGBTQ+ youth, is well documented, there are virtually no articles on the impact of various identities on long-term recovery for youth or how recovery may impact the identities youth hold. Based on the literature, it is clear that substance misuse among adolescents varies among subpopulations. There is, however, scant literature detailing the impact of a youth’s cultural intersectionality on the youth’s recovery process.

## RECOVERY-ORIENTED SYSTEMS OF CARE

Recovery-oriented systems of care (ROSC) arose out of the shortcomings of the linear, acute care model of addiction treatment. ROSC is an umbrella concept that represents the entire network of formal and informal relationships and organizations that foster individual, familial, and community recovery processes over time.^2^^(p497)^ Further explanation and elaboration of ROSC can be found elsewhere in this topic series. Although empirical evidence is mounting for adults, there is scarce literature exploring ROSC for youth. The few studies that have investigated adolescent systems have considered continuing care, mutual aid, peer groups, school programs, and technology.

A key aspect of ROSC is the continuum of care. Continuing care, frequently cited as “aftercare,” has been situated as following treatment. Like traditional treatment outcome studies, most continuing care research has studied maintenance of treatment gains. The locus of ROSC, however, has been the recovery support systems and processes themselves rather than simply indicators of treatment success. One long-term outcome study followed a treatment group, a waitlist group, and a community control group over 5.5 years post-treatment and found that involvement in continuing care among the treatment group was positively associated with improved treatment outcomes.^25^

As smartphones have taken an ever-more pervasive place in adolescent communication, they also have begun filling a role in continuing care. A recent randomized controlled trial found that voluntary recovery support provided via phone by other youths had direct and indirect effects.^26^ Continuing care was directly associated with increased involvement with pro-recovery peers and recovery management activities. It also was indirectly linked to reductions in alcohol and substance use and problems as well as increased remission. Incremental dose effects were also found—for every 10% increase in support call completion, recovery activities increased by nearly one activity.^26^ In similar fashion, Kaminer, Burleson, and Burke compared in-person and brief phone continuing care with no continuing care through a randomized design.^27^ Findings indicated that continuing care in general slowed the occurrence of post-treatment alcohol use and, for girls, maintained treatment gains; phone-based continuing care was also as effective as in-person models.^27^ More structured, manualized continuing care for adolescents, called assertive continuing care, also surfaced as an impactful model for adolescents.^15^ Although there is evidence that continuing care plays a key role in supporting recovery among adolescents, additional investigation into the moderators of both participation and effect are called for.

Another emergent youth-specific element is the incorporation of digital technology in recovery supports. Along with the previously mentioned studies utilizing phones for their financial and geographic flexibility in continuing care,^26^,^28^ Dennis and colleagues investigated and found smartphone apps to be feasible and efficacious for recovery monitoring and support among youth.^29^ The scale of benefits received from peer-based and technology-based support merits further investigation.^26^,^30^–^32^

The recovery-oriented systems of care model emphasizes communities, especially peer recovery support services. Historically, one of the most common continuing care recommendations for adolescents has been to attend mutual aid groups, such as Alcoholics Anonymous and Narcotics Anonymous.^30^ Fellowships based on a 12-step approach appear to provide a supportive social context for adolescents in recovery.^33^ Attendance and involvement in 12-step fellowships, specifically particular aspects such as meeting with a sponsor outside of meetings and verbal participation in meetings, have predicted positive recovery outcomes for adolescents over and above simple attendance, which also has been positively associated with outcomes over time.^28^,^30^,^33^ Other underlying mechanisms of 12-step benefits have included general social support and providing support to others.^28^,^34^,^35^ In combination with mutual aid, participation in religious services also was found to positively impact adolescent recovery.^28^,^36^ Expansion of youth-specific 12-step communities has been identified as a way to increase youth recovery support.^28^,^30^,^33^

ROSC, of course, is not limited to mutual aid groups. A youth model perhaps best aligned with ROSC is the alternative peer group, which began in the early 1970s. Although more evidence of effectiveness is needed, alternative peer groups (APGs) have been described in the literature as a model that integrates recovering peers, prosocial activities, and evidence-based clinical practices.^32^ Key elements of the APG model include psychosocial education, case management, social functions, community recovery support, family support, and counseling.^32^ A unique and key component of APGs is their focus on developmentally appropriate recovery support services for adolescents.

In reviewing the available evidence presented for youth recovery within ROSC, including APGs, recovery capital (RC) has surfaced as a useful frame for classification of supports and may help target specific systems or characteristics to foster youth recovery. Recovery capital is the breadth and depth of resources that persons can access to support their recovery across ecological levels.^37^ The recovery capital for adolescents model (RCAM) highlights the importance of understanding youth-specific recovery processes across four main domains of capital: human, financial, social, and community.^38^ The utility of RCAM was supported among APG participants such that RCAM identified specific recovery assets and barriers for youth as well as reflected the four recovery capital domains previously validated for adults.^31^,^32^,^38^

The review also yielded evidence of specific systems or domains of recovery capital situated within a ROSC paradigm that support youth recovery. Recovery high schools, for example, are specifically designed for students recovering from a substance use disorder. Although they have been a resource for adolescents since the late 1970s, they have only begun to be systematically empirically evaluated.^39^ A recent systematic review found only one rigorous study to date evaluating recovery high schools^40^—indicating a significant need for further investigation. These institutions of continuing support for youth are dynamic and vary widely in regards to enrollment, fiscal stability, governance, staffing, and organization; however, the tailored supports appear to benefit adolescents’ recovery and academic performance.^39^,^41^

Criminal justice institutions also present a system in which changes in practice can be more supportive for youth recovery. Evidence of the role of social support, religious service attendance, and service to others among youth who have been involved with criminal justice institutions indicated that providing a supportive recovery environment reduces the risk of relapse, incarceration, and violent crime.^34^,^35^

## FAMILIES

The family context has been identified as a significant component in the etiology and progression of adolescent alcohol and substance use for decades.^42^ Addiction has been commonly referred to as a family disease. Like most adolescent recovery research, though, the focus has been entrenched in the acute addiction treatment paradigm. Jaffe, for example, identifies family therapies as a key treatment modality for youth.^43^

The familial relationship, however, can be especially complex for adolescents seeking recovery, because they often have parents who also engage in problematic drinking or use.^16^ Despite the acknowledgement of how critical family is for adolescents seeking recovery, there remains a significant gap in the research literature focusing on recovery specifically. Possible explanations include but are not limited to the feasibility of family-based research studies. Including additional family participants in the research design increases cost and demands for methodological rigor. Future investigations into mechanisms of youth recovery are needed to better understand the familial context, as well as to situate families within the ROSC and recovery capital frames.

## NON–ABSTINENCE-BASED APPROACHES

As ROSC has emerged out of the gaps of acute care models, non–abstinence-based approaches to recovery have facilitated a new organizing paradigm surrounding multiple pathways of recovery.^5^ Although the concept of multiple pathways is not new, the exploration of harm reduction and medication-assisted treatment (MAT) and recovery is relatively recent. Shifting the focus to outcomes such as quality of life, personal relationships, life satisfaction, and improved cognition has presented new avenues for investigation and understanding treatment effectiveness. This new paradigm has particular implications for adolescents.^44^

Although the line between abstinence-based treatment and abstinence-based recovery has become less distinct over time, the lines between MAT and medication-assisted recovery have always been blurry. White said: The historical stigma attached to methadone and the broader arena of medication-assisted treatment has denied MAT patients the status of recovery and left them isolated from mainstream community life and existing in limbo between cultures of addiction and cultures of recovery. . . . At the very core of this stigma is the deeply imbedded idea that recovery from opioid addiction does not begin until the day the use of medications like methadone and buprenorphine ends. Recovery from no other chronic health condition rests on such a proposition.^45^^(p6)^

The limbo may be even more profound for adolescents. Levy and colleagues suggest MAT might be effective in the treatment of opioid use disorder for adolescents;^46^ however, Feder, Krawczyk, and Saloner found that only 2% of adolescents in treatment for heroin and opioid use received MAT, compared to 26% of adults.^47^ Beyond the long-standing philosophical issues about prescribing medications to treat AUD or other SUD, there are also concrete legal barriers in both national and state statutes that make it difficult for physicians to prescribe some medications such as methadone or buprenorphine to minors.^48^

Additional consideration is needed given the legal repercussions of harm reduction for adolescents—namely, that consumption of alcohol and cannabis is illegal for those under age 21—as well as the neurocognitive variables for the still-developing adolescent brain.^19^ Moreover, although De Sousa found that MAT, particularly disulfiram, reduced number of drinking days,^49^ Kaminer and colleagues found no evidence that harm reduction motivations for AUD yield more desirable outcomes than abstinence-based motivations among adolescents.^19^ Empirical evidence of non–abstinence-based approaches for young people is scant. Future research should explore if these approaches are safe and effective for youths.

## DISCUSSION

In a speech delivered at the UCLA/Betty Ford Institute Annual Recovery Conference in 2012, historian William White said: “People are entering recovery younger and younger, and yet little information exists about living a life in recovery that begins at age 15 or 25 rather than 45 or 55.”^2^^(p495)^ This review has shown White’s comments largely still hold. Recovery from AUD or other SUD remains a complex and challenging concept to define and thus to study, and this is even more evident for recovery that begins in adolescence. Steps have been taken, however, to distinguish recovery for people under age 18 from recovery in adulthood.

Early efforts to research youth recovery viewed it as the result of successful treatment. Recovery for adolescents was understood to be abstinence-based and usually was assumed to include some form of treatment. Studies suggested the post-treatment recovery process had a different trajectory based upon various person-level factors, including the adolescent’s cultural identity, student status, trauma history, and co-occurring disorders. Most of these studies, though, still viewed adolescent recovery through a treatment outcome lens.

The recovery-oriented systems of care approach shifted the structural and empirical locus to the recovery process itself, and it moved away from a program-level orientation to a systemic one. Although many studies of aftercare, or continuing care, still remain situated in a treatment outcome frame, the attention has gradually progressed to specific components of successful recovery for youth. Studies of adolescent ROSC, though still relatively small in number, have considered adolescent continuing care, mutual aid, peer groups, and school-based programs—as well as the impact of smartphone technology on youth recovery. Addiction also has long been understood to be a “family disease,” and there have been a few attempts to understand family systems in recovery.

Recovery increasingly has been presented as not requiring abstinence, and non–abstinence-based approaches to recovery have generated more attention in the field. The idea of multiple pathways to recovery has included paths without specialty treatment. Harm reduction and MAT approaches for youth have produced few empirical studies while getting more support philosophically. Traditional outcomes, such as relapse or even reduced days of use, have been supplanted by variables such as quality of life, personal relationships, life satisfaction, and improved cognition.

The arc of the recovery paradigm has been moving from acuteness to chronicity, from programmatic to systemic, from pathology to wellness, from exclusivity to accessibility, from homogeneity to diversity, and from selectivity to inclusivity. Diagnosis and treatment of AUD and SUD have shifted away from seeing recovery as a linear progression toward abstinence to understanding recovery moving along a continuum, which may not necessitate complete abstinence. Indeed, alcohol and other substances have even been removed from recent definitions of recovery to allow room for non–substance-related addictions—as supported by neuroscience suggesting similar brain activity for substance and non–substance-related addictions. The turn toward a “big tent” or “many roads” approach for recovery has benefits, such as mitigating stigma and facilitating healthy lives for millions of people. At the same time, the unique properties of recovery from AUD or other SUD have become harder to glean, especially as sobriety becomes less of a goal. As adolescents fundamentally differ from adults, it is essential to determine when the “big tent”/“many roads” concept—established by and for adults—will help youth and when it will not.

## FUTURE RESEARCH

A clear organizing framework is missing from the extant adolescent recovery literature. Promising work in this area includes the seminal article by Brown and Ashford around creating a “recovery science”^50^ and an article by Finch and Frieden that provides a synthesis of how classic developmental theories form a foundation for recovery high school environments and culture.^51^ It is hoped that a theoretical model will emerge from suggested future research to explain behavior change and maintenance, remission, and sustained recovery for young people.

### Harm Reduction and Medication-Assisted Recovery

As harm reduction continues to gain legitimacy as a model of recovery, more evidence is needed to understand how ongoing substance use may impact neurological as well as psychosocial development of adolescents. This review also has shown that more research is needed on how psychopharmacological drugs impact a developing brain differently from an adult brain, and how those differences implicate medication-assisted recovery. Both exploratory and effectiveness studies can guide the discussion away from passionate debates toward grounded understanding and evidence-informed program development.

### Expanding Beyond a Treatment Outcome Paradigm

The prevalence data have shown that although the number of youths with AUD or other SUD has been declining steadily over the last 2 decades, large numbers of adolescents with SUD or co-occurring disorders still do not have access to treatment and/or do not receive treatment. Although most of those youths likely do not get into recovery as adolescents, many do, and they are not being captured in the literature on recovery as a treatment outcome. One byproduct of widening the umbrella for people in recovery should be the subsequent broadening of who gets included in programs and studies.

### Disparities

Regarding the wider umbrella, adult studies of recovery have considered disparities around intersectional identities and social class in treatment and recovery. Much of the discourse about MAT, harm reduction, and abstinence-based recovery has revolved around racial disparities in the mental and behavioral health system. Youth of color “have less access to, and lower quality of, behavioral health services compared to their White counterparts.”^52^^(p22)^ These disparities and their impact on adolescent recovery trajectories need more exploration.

### Recovery Capital for Adolescents

More studies also are needed for investigating various support modalities for youth, including recovery residences, recovery high schools, alternative peer groups, mutual aid groups, and family systems, and how different combinations of components may be needed for different people and diverse populations. The nascent work on the recovery capital model for adolescents^38^ offers great promise in explaining disparities of access to certain types of recovery support, as well as which factors may benefit one young person more than another. The recovery capital model in combination with a clearer comprehension of adolescent neuroscience of addiction will better tune the field of youth recovery.

### Recovery Across the Life Span

Finally, recovery research in general needs more life course studies. Recovery begun in adolescence cannot be fully understood until adulthood. Although retrospective studies can provide some data on origination of AUD and SUD and the pathways of recovery, better precision is needed. Prospective, longitudinal, and life course research, beginning in youth and continuing at regular intervals, is the only way to fully appreciate the complex and cascading nature of recovery across the life span.

## LIMITATIONS

Neither “youth” nor “recovery” has a commonly accepted definition. Although the authors were diligent in using the literature to frame both for the purpose of this review, it is possible that defining either concept differently would have taken the review in divergent directions.

In making choices to study adolescents and the recovery process, this review did not include studies of emerging adults (ages 18 to 25) and transitional-age youth (ages 16 to 24), unless youth age 18 and younger were explicitly included in the sample. Although this allowed the authors to focus on adolescents, there may have been studies of adults whose recovery began in their youth, which were not reviewed.

Similarly, in line with the journal’s focus on alcohol, the review required alcohol and recovery to be main components in the literature search, which may have left out articles on SUD that did not explicitly mention alcohol. The language used in extant literature guided the findings. In studies related to recovery and young people, AUD and SUD often were discussed in one category instead of referencing alcohol and various substances in their own capacity. Hennessy and Fisher provide an example of how future studies could review literature related to broader substance use and recovery among young people.^53^

Though population effects are considered here, the review does not fully explore the diversity of adolescent recovery experiences based on intersecting identities or social class. This is due in large part to the lack of diversity in both adolescent recovery support programs and in research studies.

Finally, while using this topic series’ own categorizations as an organizing frame allowed for conceptual consistency, it can be acknowledged that different reviewers may have arrived at a different heuristic typology. No review of adolescent recovery at this stage should be considered definitive, and this review is no exception. Rather, the intent was that this integrative review would be well designed, thorough, and an accurate representation of the field to date.

## CONCLUSION

As the recovery movement has become established and access to recovery has broadened, the need to explain and study how the concept of recovery pertains specifically to adolescents has increased. This integrative review considered studies of youth and recovery across (1) treatment outcomes, (2) special emphasis populations, (3) recovery-oriented systems of care, (4) families, and (5) non–abstinence-based approaches. Although this review found that the literature on adolescent recovery has grown, the authors make the following recommendations:

- More research is needed about the impact and effectiveness of medication-assisted recovery and harm reduction.
- The field of adolescent recovery needs to widen its scope of practice and research beyond youth who have received treatment to include those who have not received treatment due to personal choice or societal disparities.
- The literature would benefit from more prospective and life course research.

Research must not lose sight of the unique properties of either adolescent development or recovery from alcohol or other substance-related disorders, and there is great promise in the recovery capital model for adolescents and the neuroscience of addiction to provide more precision and direction to the field of recovery and youth.

## Figures and Tables

**Table 1:** References Identified in Literature Search (N = 39)

Main Topic	Reference Number	Author	Year
**Treatment Outcomes**			
	8	Brown et al.	2001
	9	Myers, Brown, and Mott	1993
	10	Brown and Ramo	2006
	11	Latimer et al.	2000
	12	Tapert et al.	1999
	13	Chung et al.	2015
	14	Cornelius et al.	2003
	15	Kaminer and Godley	2010
	16	Cavaiola, Schiff, and Kane-Cavaiola	1990
	17	Maisto et al.	2003
	18	Chung et al.	2005
	19	Kaminer et al.	2018
**Special Emphasis Populations**			
	20	McCarthy et al.	2005
	21	Sterling et al.	2009
	22	Pagano et al.	2015
	23	Krentzman et al.	2012
	24	Winward et al.	2014
**Recovery-Oriented Systems of Care**			
	25	Winters et al.	2007
	26	Godley et al.	2019
	27	Kaminer, Burleson, and Burke	2008
	15	Kaminer and Godley	2010
	28	Chi et al.	2009
	29	Dennis et al.	2015
	30	Kelly and Urbanoski	2012
	31	Nash, Hennessy, and Collier	2019
	32	Nash and Collier	2016
	33	Nash	2020
	34	Johnson et al.	2016
	35	Johnson et al.	2018
	36	Pullen et al.	1999
	37	Cloud and Granfield	2008
	38	Hennessy, Cristello, and Kelly	2019
	39	Finch, Moberg, and Krupp	2014
	40	Hennessy et al.	2018
	41	Finch et al.	2018
**Families**			
	42	Stewart and Brown	1993
	43	Jaffe	2002
**Non–Abstinence-Based Approaches**			
	44	Marlatt and Witkiewitz	2002
	49	De Sousa	2014
	19	Kaminer et al.	2018
